# 981 PI Project: Reduction of Hospital-Acquired Infection Transference Through Optimization of Ultraviolet-C System Placement in BICU

**DOI:** 10.1093/jbcr/iraf019.512

**Published:** 2025-04-01

**Authors:** Lia Applegarth, Emily Allred, Zachary Ditzig, Tait Olaveson

**Affiliations:** Eastern Idaho Regional Medical Center; Eastern Idaho Regional Medical Center; The Burn Center at Eastern Idaho Regional Medical Center; The Burn Center at Eastern Idaho Regional Medical Center

## Abstract

**Introduction:**

Hospital-acquired infections (HAIs) are a major concern in the healthcare field. In the Burn Intensive Care Unit (BICU), patient’s infection is a leading cause of morbidity and mortality in burn patients. Additionally, HAI transference from patient-to-patient in the BICU is a well documented occurrence. In this process improvement (PI) project, ultraviolet-C (UVC) systems were implemented and assessed, leading to a change in terminal cleaning policy and optimization of disinfection. It is hypothesized that this optimization of UVC system placement in terminal cleaning led to a decrease in patient-to-patient HAI transference.

**Methods:**

A framework was developed to standardize and optimize the placement of the UVC systems in the BICU. Shape and size of BICU rooms were the main considerations in determining the placements of the UVC systems such that BICU rooms would get complete and adequate dosage of UVC. Efficacy of this framework was measured by tracking the incidence of suspected cross-contamination HAIs before and after implementation.

**Results:**

Development of optimal marking placements for UVC systems in the BICU led to optimized decontamination in the terminal cleaning of the rooms. After implementation of the standardized terminal cleaning policy, suspected HAIs from cross-contamination in the BICU decreased from 3 cases the previous year to 0 the following year.

**Conclusions:**

Standardizing UVC system placement ensures complete coverage and proper UVC disinfection of the BICU rooms as well as decreasing the incidence of potential environmental cross-contamination, transference, and HAIs. Complete and proper UVC decontamination of any hospital room is important and should be ascertained, especially in medical surgical ICUs and other units where infection transference risk is high.

**Applicability of Research to Practice:**

Standardizing the placement of decontamination systems to optimize cleaning may help reduce cross-contamination in BICU rooms.

**Funding for the Study:**

N/A

